# Standardizing an approach to the evaluation of implementation science proposals

**DOI:** 10.1186/s13012-018-0770-5

**Published:** 2018-05-29

**Authors:** Erika L. Crable, Dea Biancarelli, Allan J. Walkey, Caitlin G. Allen, Enola K. Proctor, Mari-Lynn Drainoni

**Affiliations:** 1 Evans Center for Implementation and Improvement Sciences, Boston University School of Medicine, 88 East Newton Street, Vose 216, Boston, MA 02118 USA; 2 Department of Health Law, Policy & Management, Boston University School of Public Health, Boston, MA USA; 3 Section of Pulmonary, Allergy, and Critical Care Medicine, Department of Medicine, Boston University School of Medicine, Boston, MA USA; 4 Behavioral Sciences and Health Education Department, Rollins School of Public Health, Emory University, Atlanta, GA USA; 5 Center for Mental Health Services Research, The Brown School at Washington University in St. Louis, St. Louis, MO USA; 6 Section of Infectious Diseases, Department of Medicine, Boston University School of Medicine, Boston, MA USA; 7 Center for Healthcare Organization and Implementation Research, Edith Nourse Rogers Memorial VA Hospital, Bedford, MA USA

**Keywords:** Implementation research, Improvement research, Grant writing, Grant scoring

## Abstract

**Background:**

The fields of implementation and improvement sciences have experienced rapid growth in recent years. However, research that seeks to inform health care change may have difficulty translating core components of implementation and improvement sciences within the traditional paradigms used to evaluate efficacy and effectiveness research. A review of implementation and improvement sciences grant proposals within an academic medical center using a traditional National Institutes of Health framework highlighted the need for tools that could assist investigators and reviewers in describing and evaluating proposed implementation and improvement sciences research.

**Methods:**

We operationalized existing recommendations for writing implementation science proposals as the *ImplemeNtation and Improvement Science Proposals Evaluation CriTeria* (INSPECT) scoring system. The resulting system was applied to pilot grants submitted to a call for implementation and improvement science proposals at an academic medical center. We evaluated the reliability of the INSPECT system using Krippendorff’s alpha coefficients and explored the utility of the INSPECT system to characterize common deficiencies in implementation research proposals.

**Results:**

We scored 30 research proposals using the INSPECT system. Proposals received a median cumulative score of 7 out of a possible score of 30. Across individual elements of INSPECT, proposals scored highest for criteria rating evidence of a care or quality gap. Proposals generally performed poorly on all other criteria. Most proposals received scores of 0 for criteria identifying an evidence-based practice or treatment (50%), conceptual model and theoretical justification (70%), setting’s readiness to adopt new services/treatment/programs (54%), implementation strategy/process (67%), and measurement and analysis (70%). Inter-coder reliability testing showed excellent reliability (Krippendorff’s alpha coefficient 0.88) for the application of the scoring system overall and demonstrated reliability scores ranging from 0.77 to 0.99 for individual elements.

**Conclusions:**

The INSPECT scoring system presents a new scoring criteria with a high degree of inter-rater reliability and utility for evaluating the quality of implementation and improvement sciences grant proposals.

## Background

The recognition that experimental efficacy studies alone are insufficient to improve public health [1] has led to the rapid expansion of the fields of implementation and improvement sciences [2–5]. However, studies that aim to identify strategies that facilitate adoption, sustainability, and scalability of evidence may not translate well within traditional efficacy and effectiveness research paradigms [6].

The need for new tools to aid investigators and research stakeholders in implementation science became clear during evaluation of grant submissions to the Evans Center for Implementation and Improvement Sciences (CIIS) at Boston University. CIIS was established in 2016 to promote scientific rigor in new and ongoing projects aimed at increasing the use of evidence and improving patient outcomes within an urban, academic, safety net medical center. As part of CIIS’s goal to foster rigorous implementation and improvement methods, CIIS established a call for pilot grant applications for implementation and improvement sciences [7]. Proposals were peer-reviewed using traditional National Institutes of Health (NIH) scoring criteria [8]. Through two cycles of grant applications, proposal reviewers identified a need for improved evaluation criteria capable of identifying specific strengths and weaknesses in order to rate the potential impact of implementation and/or improvement study designs.

We describe the development and evaluation of *ImplemeNtation and Improvement Science Proposal Evaluation CriTeria (INSPECT)*: a tool for the standardized evaluation of implementation and improvement research proposals. The INSPECT tool seeks to operationalize criteria proposed by Proctor et al. as “key ingredients” that constitute a well-crafted implementation science proposal, which operate within the NIH proposal scoring framework [6].

## Methods

### Assessment of need

CIIS released requests for pilot grant applications focused on implementation and improvement sciences in April 2016 and April 2017 [7]. The request for applications described an opportunity for investigators to receive up to $15,000 for innovative implementation and improvement sciences research on any topic related to improving the processes and outcomes of health care delivery in safety net settings. CIIS funds pilot grants with the goal of providing investigators with the opportunity to obtain preliminary data for further research. Proposals were required to include a specific aims page and a three-page research plan structured within the traditional NIH framework with subheadings for significance, innovation, approach, environment, and research team. The NIH framework was required because it corresponds with the grant proposal structure required by the NIH. A study budget and justification as well as research team biographical sketches were required with no page limit restrictions. CIIS received 30 pilot grant applications covering a broad array of content areas, such as smoking cessation, hepatitis C, diabetes, cancer, and neonatal abstinence syndrome.

Six researchers with experience in implementation and improvement sciences served as grant reviewers. Four reviewers scored each proposal. Reviewers evaluated the quality of pilot study proposals, assigning numerical scores from 1 to 9 (1 = exceptional, 9 = poor) for each of the NIH criteria (significance, innovation, investigators, approach, environment, overall impact) [8]. CIIS elected to use the NIH criteria to evaluate the pilot grant applications because the criteria are those used by the NIH peer review systems to evaluate the scientific and technical merit of grant proposals. The CIIS grant review team held a “study section” to review and discuss the proposals. However, during that meeting, reviewers provided feedback that the NIH evaluation criteria, based in the traditional efficacy and effectiveness research paradigm, did not offer sufficient guidance for evaluating implementation and improvement science proposals, nor did it provide enough specificity for the proposal writers who are less experienced in implementation research. Grant reviewers requested new proposal evaluation criteria that would better inform score decisions and feedback to proposal writers on specific aspects of implementation science including measuring the strength of implementation study design, strategy, feasibility, and relevance.

Despite the challenges of using the traditional NIH evaluation criteria, the review panel used those criteria to score all of the grants received during the first 2 years of proposal requests. CIIS pilot grant funding was awarded to applications that received the lowest (best) scores under the NIH criteria and received positive feedback from the review panel.

The request for more explicit implementation science evaluation criteria prompted the CIIS research team to conduct a qualitative needs assessment of all 30 pilot study applications in order to determine how the proposals described study designs, implementation strategies, and other aspects of proposed implementation and improvement research. Three members of the CIIS research team (MLD, AJW, DB) independently open-coded pilot proposals to identify properties related to core implementation science concepts or efficacy and effectiveness research [9]. The team identified common themes in the proposals, including an emphasis on efficacy hypotheses, descriptions of untested interventions, and the absence of implementation strategies and conceptual frameworks. The consistent lack of features identified as important aspects of implementation science reinforced the need for criteria that specifically addressed implementation science approaches to guide both proposal preparation and evaluation.

### Operationalizing scoring criteria

We identified Proctor et al.’s “ten key ingredients” for writing implementation research proposals [6] as an appropriate framework to guide and evaluate proposals. We operationalized the “ingredients” into a scoring system. To construct the scoring system, a four-point scale (0–3) was created for each element. In general, a score of 3 was given for an element if all of the criteria requirements for the element were fully met; a score of 2 was given if the criteria were somewhat, but not fully addressed; a score of 1 was given if the ingredient was mentioned but not operationalized in the proposal or linked to the rest of the study; and a score of 0 was given if the element was not addressed at all in the proposal. Table 1 illustrates the INSPECT scoring system for the 10 items, in which proposals receive one score for each of the 10 ingredients, for a cumulative score between 0 and 30.

**Table 1:** Implementation and Improvement Science Proposal Evaluation Criteria

Criteria	Score			
	0	1	2	3
The care or quality gap	• No care/quality gap is defined; an issue may be presented, but it is not described as a gap in quality or care • No information or lack of clarity in the information cited about the potential for improvement or the impact of the proposed implementation and/or improvement science study • Proposed implementation and/or improvement science study is not linked to a safety net setting	• Unclearly defined care/quality gap is poorly supported with inappropriate/inadequate/irrelevant local setting data (i.e., evidence of chart review or other preliminary data) or citations from the literature • Insufficient information about the potential for improvement or the impact of the proposed implementation and/or improvement science study • Proposed implementation and/or improvement science study does not explicitly link to a safety net setting	• Defined care/quality gap is supported by either local setting data (i.e., evidence of chart review or other preliminary data) or citations from the literature • Adequate information about the potential for improvement, but would benefit from further specification • Proposed implementation and/or improvement science study links to a safety net setting but may need further clarification	• Clearly defined quality gap is supported by local setting data (i.e., evidence of chart review or other preliminary data) and appropriate citations from the literature • Explicit, well thought out description of the potential for improvement • Proposed implementation and/or improvement study is clearly linked to a safety net setting
The evidence-based treatment to be implemented	• No evidence-based or evidence-informed intervention is identified or the intervention justification/background is based on zero/inappropriate/inadequate citations from literature • Lack of clarity about why the intervention was chosen for the study setting • Unclear what effect the intervention will have on the selected safety net setting (or study is not based in safety net setting)	• Some literature is cited to provide evidence of limited prior efficacy studies concerning the planned intervention, meeting “evidence-informed” rather than “evidence-based” criteria • Limited justification about why the intervention was chosen for the study setting and/or justification is based on desire to document efficacy of the proposed evidence-informed practice • Insufficient information describing what effect the intervention will have on the selected safety net setting	• Sufficient literature is cited demonstrating evidence of prior efficacy studies using the intervention to meet either “evidence-informed” or “evidence-based” criteria • If the intervention is “evidence-informed,” the innovative use of said intervention in the study setting is compelling enough to consider, or there is appropriate justification about why the evidence-based intervention was chosen for the study setting, and the goal is not based on developing efficacy of the said evidence-informed practice • Adequate information describing what effect the intervention will have on the selected safety net setting, but may need further clarification	• Clearly discusses evidence of prior efficacy studies concerning the planned intervention, meeting “evidence-based” rather than “evidence-informed” criteria • Explicit, well thought-out rationale for implementing the intervention in the selected safety net setting including the potential effect it will have on that setting
Conceptual model and theoretical justification	• No conceptual model, framework, or other theoretical grounding is discussed • Some conceptual model is cited but its basis and constructs are irrelevant to study objectives and/or the study setting	• A conceptual model, framework, or other theoretical grounding is mentioned, but not linked to the study objectives, hypotheses, and measures • The chosen conceptual model, framework, or other theoretical grounding may be appropriate for the intervention, but the rationale is not clearly supported with citations from the literature	• A conceptual model, framework, or other theoretical grounding is linked in some capacity to the study objectives, hypotheses, and measures, but may need additional clarification • The chosen conceptual model, framework, or other theoretical grounding is appropriate for the intervention /implementation strategies as evidenced by a well-defined rationale with adequate citationsfrom the literature, but would still benefit from further specificity	• An implementation and/or improvement science-specific conceptual model or framework is clearly described, with theoretical constructions explicitly described within the proposed setting, population, and intervention contexts • The implementation and/or improvement science-specific conceptual model or framework is used to frame the proposed study in all aspects including the study questions, aims/objectives, hypotheses, process, and outcome measures • Some discussion may refer and describe how study findings would build upon or otherwise contribute to theory or the larger implementation and/or improvement science fields
Stakeholder priorities, engagement in change	• Zero or extremely limited description of who the stakeholders are or what their preferences and priorities are around the proposed intervention • No evidence of stakeholder analysis planning, or basic information gathering is discussed in relation to how the applicant developed the implementation strategies	• Limited description of who the stakeholders are, with some key players missing from consideration • Limited understanding of stakeholder priorities and concerns related to the intervention is demonstrated by easily identified potential issues that are not discussed in the application, or no evidence of stakeholder analysis planning is discussed • Zero or very limited mention of involving stakeholders in the conceptual design of the intervention, and/or consideration of the implementation strategies, process, or outcomes • No clear agreement or collaboration between the stakeholders and the applicant is explained	• Sufficient description of who all of the identifiable stakeholders are • Clear understanding of stakeholder concerns related to the intervention as evidenced by a stakeholder analysis plan that describes how the applicant will collect at least some information on stakeholders interests, interrelations, influences, preferences, or/and priorities • Somewhat unclear description of how stakeholders were involved in the conceptual design of the intervention, and/or consideration of the implementation strategies, process, or outcomes • Some type of agreement or collaboration between the stakeholders and the applicant is explained but supporting evidence is limited	• Comprehensive description of who all of the identifiable stakeholders are • Clear understanding of stakeholder concerns related to the intervention as evidenced by a stakeholder analysis plan that describes how the applicant will collect comprehensive information on stakeholders interests, interrelations, influences, preferences, and priorities • Detailed description of how stakeholders were involved in the conceptual design of the intervention and in considering the implementation strategies, process, and outcomes • An explicit agreement (such as a memorandum of understanding) or evidence of collaboration between the stakeholders and the applicant that is explained with relevance to the proposed study process and how findings will be communicated
Settings readiness to adopt new services/treatment/programs	• Zero or very limited rationale/interest for implementing the proposed intervention is discussed • No information on the study setting’s capacity or readiness for implementation • No information on how those in the study setting who are opposed to change will be involved with or have their concerns addressed by study processes or components	• Some description of the setting’s interest in the proposed intervention • Incomplete or unclear description of how the setting will be assessed for capacity and/or readiness for implementation including which methods and tools will be used, or there is a limited description of organizational/political culture and potential contextual barriers or facilitators • May include a brief discussion on how those opposed to change in the study setting will be involved with or have their concerns addressed by study processes or components • May not include evidence of support (e.g., letters) from the study setting that address how the proposed study aligns with the organization’s priorities/policies	• Clearly describes the setting’s interest and rationale for the proposed intervention • Clearly describes how the setting will be assessed for capacity and readiness for implementation including which methods, scales, or other tools will be used • Thoroughly describes the potential influence of organizational/political culture, and potential contextual barriers or facilitators • May include strategies for how those opposed to change in the study setting will be involved with or have their concerns addressed by study processes or components • May not include evidence of support (e.g., letters) from the study setting that address how the proposed study aligns with the organization’s priorities/policies	• Explicitly describes preliminary data on the assessed organizational and political capacity and readiness for implementation (assessment completed prior to application/pilot) • Preliminary capacity and readiness assessments were completed using a scale with established validity and reliability, or a scale that has undergone some validity and reliability testing • May include strategies for how those opposed to change in the study setting will be involved with or have their concerns addressed by study processes or components • Evidence of support (e.g., letters) from the study setting that address how the proposed study aligns with the organization’s priorities/policies
Implementation strategy/process	• No implementation strategies are identified • Intervention may be incorrectly described as an implementation strategy	• Implementation strategies are not clearly distinguished from the intervention • Unclear implementation strategies are not theoretically justified and/or do not match with the stated aims/setting/outcome measures of the proposed study • Limited description linking the implementation strategies to the stated aims/setting/outcome measures of the proposed study with no plan for how strategies will be observed or tested • Implementation strategies may be unrealistic given the pilot timeline and/or budget constraints	• Implementation strategies are clearly distinguished from the intervention • Some theoretical justification of the implementation strategies • Clearly describes how implementation strategies link to the stated aims/setting/outcome measures of the proposed study • More description is needed to clearly understand how implementation strategies will be observed or empirically tested • Implementation strategies are mostly feasible given the pilot study timeline and budget constraints	• Explicitly describes and theoretically justifies the implementation strategies • Explicitly describes how implementation strategies link to the stated aims/setting/outcome measures of the proposed study • Explicitly describes how implementation strategies will be observed or empirically tested • Implementation strategies are feasible given the pilot study timeline and budget constraints
Team experience with setting, treatment, and implementation process	• Only the principal investigator’s skills are described • No additional information, biographical sketches, resumes/CVs are provided beyond the principal investigator	• It is unclear how the team experience relates to the study setting, treatment, and/or processes • Staffing plan may not facilitate successful study completion without significant support from CIIS • Team experience is uniform and does not offer multidisciplinary skills or perspective to the proposed study	• Team description, biographical sketches, resumes/CVs depict a multidisciplinary skillset relevant to the proposed study setting, treatment, processes, and other needs • Staffing plan facilitates successful study completion, with some support from CIIS likely necessary • No description of the research environment strengths including resources and/or infrastructure • If principal investigator is considered junior or early career or novice to implementation science, it is unclear what senior leadership outside of CIIS will be available for mentoring and/or consultation	• Clearly describes how team experience relates to the study setting, treatment, and processes • Team description, biographical sketches, resumes/CVs depict a multidisciplinary skillset relevant to the proposed study setting, treatment, processes, and other needs • Staffing plan facilitates successful study completion without necessitating CIIS support • Clearly describes strengths of the research environment including resources and infrastructure • If principal investigator is considered junior or early career or novice to implementation science, senior leadership outside of CIIS has been identified to support study completion with mentoring and/or consultation
Feasibility of proposed research design and methods	• The proposed study includes methods, interventions, and other components that are beyond the scope of a pilot study and/or inappropriate for a pilot study • A budget and/or timeline are not included or are unrealistic • Potential barriers to implementation are not described or are insurmountable	• The proposed study includes methods, interventions, and other components that may be challenging to accomplish • The budget and/or timeline are not included or unrealistic • Potential barriers to implementation are not clearly described or are insurmountable	• The proposed study includes appropriate methods, interventions, and other components that are likely achievable as a pilot study • The budget and/or timeline may need some revising • Potential barriers to implementation are clearly described but may lack clear description of how those barriers will be overcome	• The proposed study includes appropriate methods, interventions, and other components that are achievable as a pilot study and are justified against potential alternatives • The budget and timeline are appropriate • Potential barriers to implementation are clearly identified with potential plans to overcome those barriers
Measurement and analysis section	• Outcomes described are not implementation or improvement science-related • Outcomes are not linked to the proposed study aims • The unit of analysis is inappropriate for the proposed study • No measurement and/or data analysis plan are included to describe how variables and outcomes will be measured	• Outcomes described are implementation and/or improvement science-related • Outcomes are unclearly linked to the proposed study aims • The unit of analysis is appropriate for the proposed study • Measurement and/or data analysis plans do not clearly describe how all variables and outcomes will be measured, or plans are inappropriate for the proposed study	• Outcomes described are implementation and/or improvement science-related • Outcomes are clearly linked to the proposed study aims • The unit of analysis is appropriate for the proposed study • Measurement and/or data analytic plans describe how all variables and outcomes will be measured and is appropriate for the proposed study, but linkage to the theoretical model is unclear	• Outcomes described are implementation and/or improvement science-related • Outcomes are clearly linked to the proposed study aims • The unit of analysis is appropriate for the proposed study • Measurement and data analytic plans robustly describe how all variables and outcomes will be measured and are appropriate for the proposed study through a clear theoretical justification
Policy/funding environment; leverage of support for sustaining change	• No acknowledgement of the internal/external policy trends and/or funding environment for the propose study is included • Zero or limited discussion of the potential impact of the intervention is included • Zero or limited discussion of disseminating study findings is included	• The internal/external policy trends and/or funding environment are discussed but additional clarification is needed • The potential impact of the intervention is not linked to the policy and/or funding context and may not be relevant to a safety net setting • The dissemination plan for study findings does not clearly indicate a contribution will be made to the broader policy level and safety net setting	• The internal/external policy trends and/or funding environment are clearly described • The potential impact of the intervention is linked to relevant policies and funding issues associated with a safety net setting but may need further explanation • The dissemination plan for study findings indicates a contribution will be made to the broader policy level and safety net setting, but what contribution and how it will be achieved is unclear	• The internal/external policy trends and/or funding environment are clearly described • Potential impact of the intervention is explicitly linked to relevant policies and funding issues associated with a safety net setting • The dissemination plan for study findings indicates what and how a contribution will be made to the broader policy level and safety net setting

### Testing INSPECT

We used the pilot study proposals submitted to CIIS to develop and evaluate the utility and reliability of the INSPECT scoring system. Initially, two research team members (ELC, DB) independently applied the 10-element criteria to 7 of the 30 pilot grant proposals. Four team members (MLD, AJW, ELC, DB) then met to discuss these initial results and achieve consensus on the scoring criteria. Two team members (ELC, DB) then independently scored the remaining 23 pilot study applications using the revised scoring system. Both reviewers recorded brief justifications for each of the ten scores assigned to individual study proposals. The two coders (ELC, DB) then met to compare scores, share scoring justifications, and determine the final item-specific scores for each proposal using group consensus.

Inter-coder reliability with the scoring protocol was measured using Krippendorff’s alpha to assess observed and expected disagreement between the two coders’ initial individual item scores [10, 11]. An alpha coefficient of 0.70 was deemed a priori *as* the lowest acceptable level of agreement to establish reliability of the new scoring protocol [10, 11]. Frequency analyses were conducted to determine the distribution of final element-specific scores (0–3) across all proposals. We calculated a correlation coefficient to assess the association between proposal scores assigned using the NIH framework and scores assigned using INSPECT. All calculations were performed in R version 3.3.2 [12].

## Results

Iterative review of the 30 research proposals using Proctor et al.’s “ten key ingredients” resulted in the development and testing of the INSPECT system for assessing implementation and improvement science proposals.

**Fig. 1:**
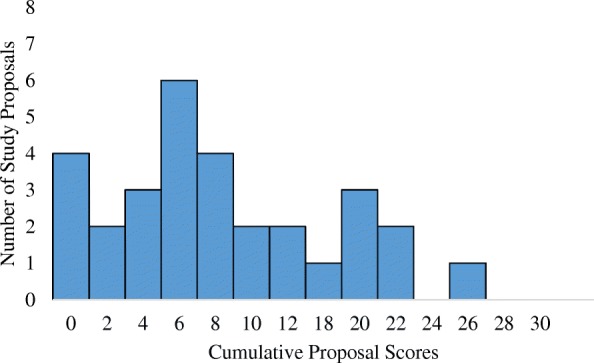
Distribution of cumulative proposal scores assigned using ImplemeNtation and Improvement Science Proposal Evaluation CriTeria (INSPECT)

Figure 1 displays the skewed right distribution of cumulative proposal scores, with most proposals receiving low overall scores. Out of a possible cumulative score of 30, proposals had a median score of 7 (IQR 3.3–11.8).

Table 2 presents the distribution of cumulative and item-specific scores assigned to proposals using the INSPECT criteria. Across individual elements, proposals scored highest for criteria describing care/quality gaps in health services. Thirty-six percent of proposals received the maximum score of 3 for meeting all care or care or quality gap element requirements, including using local setting data to support the existence of a gap, including an explicit description of the potential for improvement, and linking the proposed research to funding priorities (i.e., safety net setting).

**Table 2:** Distribution of ImplemeNtation and Improvement Science Proposal Evaluation CriTeria (INSPECT) Scores

Cumulative Proposal Scores					
Proposals evaluated: *n* = 30, Median score 7 (IQR 3.3–11.8)					
Individual Item Scores					
INSPECT Items	Rating Scale				Krippendorff’s Alpha
	0	1	2	3	
	(*N* = 150)	(*N* = 74)	(*N* = 47)	(*N* = 29)	(0.88)
The care gap or quality gap	7 (23%)	6 (20%)	6 (20%)	11 (36%)	0.84
The evidence-based treatment to be implemented	15 (50%)	9 (30%)	2 (7%)	4 (13%)	0.77
Conceptual model and heoretical justification	21 (70%)	4 (13%)	3 (10%)	2 (7%)	0.99
Stakeholder priorities, engagement in change	13 (43%)	9 (30%)	7 (23%)	1 (3%)	0.88
Setting’s readiness to adopt new services/treatment/programs	16 (53%)	7 (23%)	6 (20%)	1 (3%)	0.96
Implementation strategy/process	20 (67%)	7 (23%)	1 (3%)	2 (7%)	0.84
Team experience with setting, treatment, and implementation process	13 (43%)	5 (17%)	8 (27%)	4 (13%)	0.96
Feasibility of proposed research design and methods	13 (43%)	11 (37%)	6 (20%)	0 (0%)	0.84
Measurement and analysis	21 (70%)	4 (13%)	3 (10%)	2 (7%)	0.78
Policy/funding environment; leverage or support for sustaining change	11 (37%)	12 (40%)	5 (17%)	2 (7%)	0.77

Proposals generally scored poorly for other criteria. As shown in Table 2, most study proposals received scores of 0 in the categories of evidence-based treatment to be implemented (50%), conceptual model and theoretical justification (70%), setting’s readiness to adopt new services/treatment/programs (53%), implementation strategy/process (67%), and measurement and analysis (70%). For example, reviewers gave scores of 0 for the “evidence-based intervention to be implemented” element because the intervention was not evidence-based and the project sought to establish efficacy, rather than to examine uptake of an established evidence-based practice. Similarly, proposals that only sought to study effectiveness and did not assess any implementation outcomes [13] (e.g., adoption, fidelity) received scores of 0 for “measurement and analysis.” None of the study proposals primarily aiming to assess effectiveness outcomes expressed the dual research intent of a hybrid design. Scores of 0 for other categories were given when applications lacked any description relevant to the category, such as no conceptual model, no implementation strategy, or no research team skills revenant to implementation or improvement science.

Table 2 displays the assessed rates of inter-coder reliability in applying INSPECT to the 30 pilot study proposals. An overall alpha coefficient of 0.88 was observed between the coders. Rates of inter-coder reliability in applying each of the 10 items to the proposals ranged from 0.77 to 0.99, all above the 0.70 reliability threshold.

Additionally, we observed a moderate inverse correlation (*r* = − 0.62, *p* < 0.01) between the proposal scores initially assigned using the NIH framework and the scores assigned using INSPECT.

## Discussion

We developed a reliable proposal scoring system that operationalizes Proctor et al.’s “ten key ingredients” for writing an implementation research grant [6]. Previous research analyzing peer-review grant processes has highlighted a need to improve scoring agreement between peer reviewers [14]. High levels of disagreement in assessors’ interpretation of grant scoring criteria result in unreliable peer-review processes and funding decisions based more on chance than scientific merit [14]. Measuring rates of inter-rater reliability are a standard approach for evaluating the utility of existing proposal scoring criteria and assessing efforts to improve the criteria [15, 16]. Application of the INSPECT system demonstrated high inter-rater reliability overall and within each of the 10 items. The high degree of reliability measured for INSPECT may be related to the specificity of its design as an implementation and improvement science scoring criteria. A review of scoring rubrics reported in the scientific literature suggests that topic-focused criteria contribute to increased scoring reliability [17]. Additionally, the moderate correlation between scores assigned using the NIH framework and scores assigned using INSPECT suggests validity of the INSPECT criteria in evaluating proposal quality. Proctor et al.’s “ten key ingredients” for grant writers were developed to map onto the existing NIH criteria. Our operationalized version of the ingredients as scoring criteria demonstrated that proposals that scored poorly under NIH criteria also scored poorly under INSPECT.

Applying the INSPECT system to proposed implementation and improvement science research at an academic medical center improved proposal reviewers’ ability to identify specific strengths and weaknesses in implementation approach. Overall, proposals only received high scores for identifying the care gap or quality gap. Since efficacy and implementation or improvement research may use similar techniques to establish the significance of the study questions [18], proposals may score well on describing the quality gap, even if they later described efficacy hypotheses that received overall low scores from the INSPECT system. Further studies should explore techniques for describing care and quality gaps that highlight implementation or improvement research questions.

Consistently low scores in four areas—defining the evidence-based treatment to be implemented, conceptual model and theoretical justification, setting’s readiness to adopt new programs, and measurement and analysis—suggest that many investigators seeking to conduct implementation research may have misconceptions about the fundamental goals of this field. One misconception may relate to a sole focus on evaluating an intervention’s effectiveness rather than studying the processes and outcomes of implementation strategies. The majority of study proposals evaluated using INSPECT neither aimed to improve uptake of any evidence-based practice nor included any implementation measures such as acceptability, adoption, feasibility, fidelity, penetration, or sustainability [19]. Inadequate and inconsistent descriptions of implementation strategies and outcomes represent major challenges to overall implementation study success [20]. In addition to guidance provided by the INSPECT criteria, recent efforts to develop implementation study reporting standards [21] may assist proposal writers in describing planned research.

Several proposals addressed treatments or practices with low evidence for the potential to improve healthcare. Although hybrid studies, which study both effectiveness and implementation outcomes, are practical approaches to establishing the effectiveness of evidence-informed practices while measuring implementation efforts [18], none of the study proposals expressed this dual research intent or were conceived as hybrid designs.

Our findings also suggest low familiarity with and use of resources to evaluate of the strength of evidence (such as the Grading Quality of Evidence and Strength of Recommendations system [22] and the Strength of Recommendation Taxonomy grading scale [23]) for implementation science research. A more systematic evaluation of the strength of evidence [24–27] necessary to warrant implementation efforts may help to differentiate implementation science from efficacy or effectiveness research and improve understanding of the utility hybrid studies offer [28].

Expanding access to implementation science training in universities as part of the core health services research curriculum and enhancing access to professional development opportunities that focus on conceptual and methodological implementation skills in a content agnostic way would aid in building capacity for the next generation of implementation science researchers. Additionally, training programs provide an opportunity to provide guidance on both writing and evaluating the quality of implementation science grant applications.

Strengths of our results include that application of INSPECT to study proposals submitted by investigators with a wide range of implementation and improvement science-specific experience, and covering a variety of content areas. However, our results are limited in that they characterize one academic institution’s familiarity with implementation and improvement science research and the INSPECT system requires validation in other settings and over a broader range of proposal ratings. Additionally, we measured a high degree of inter-rater reliability for INSPECT when it was applied to a sample of low-scoring proposals. INSPECT’s inter-rater reliability may decrease when applied to a sample of higher quality proposals, and reviewers are required to discriminate between gradations of quality (i.e., scores of 1–3) rather than mostly scoring the absence of key items (i.e., scores of 0). Future research should test the validity of INSPECT by comparing INSPECT-assigned scores to ratings assigned to approved proposals by the NIH Dissemination and Implementation Research in Health study section. Future research should also assess the relationship between INSPECT score assignments and successful study completion to determine the utility of INSPECT as a mechanism for ensuring the quality and impact of funded research. To aid in these prospective research efforts, forthcoming proposal calls from CIIS will specifically use INSPECT as the proposal evaluation criteria.

Although multiple tools exist to aid researchers in writing implementation science proposals [6, 29, 30], few resources exist to support grant reviewers. This study identified additional functionality of Proctor et al.’s “ten key ingredients” as a guide for writers by developing it into a detailed checklist for proposal reviewers. The current research makes a substantive contribution to implementation and improvement sciences by demonstrating the utility and reliability of a new tool designed to aid grant reviewers in identifying high-quality research.

## Conclusion

In conclusion, we operationalized an implementation and improvement research-specific scoring system to provide guidance for proposal writers and grant reviewers. We demonstrated the utility and reliability of the new INSPECT scoring systems in evaluating the quality of implementation and improvement sciences research proposed at one academic medical center. The prevalence of low scores across the majority of INSPECT criteria suggests a need to promote education about the goals of implementation and improvement science, including the conceptual and methodological distinctions from efficacy and effectiveness research.

## Abbreviations

CIIS: Center for Implementation and Improvement Sciences
INSPECT: Implementation and Improvement Science Proposal Evaluation Criteria
NIH: National Institutes of Health

## References

[1] Glasgow RE, Lichtenstein E, Marcus AC (2003). Why don’t we see more translation of health promotion research to practice? Rethinking the efficacy-to-effectiveness transition. Am J Public Health.

[2] Neta G, Sanchez MA, Chambers DA, Phillips SM, Leyva B, Cynkin L (2015). Implementation science in cancer prevention and control: a decade of grant funding by the National Cancer Institute and future directions. Implement Sci.

[3] Purtle J, Peters R, Brownson RC. A review of policy dissemination and implementation research funded by the National Institutes of Health, 2007–2014. Implement Sci. 2015;11(1) 10.1186/s13012-015-0367-1. 10.1186/s13012-015-0367-1 PMC4700744 26727969

[4] Tinkle M, Kimball R, Haozous EA, Shuster G, Meize-Grochowski R (2013). Dissemination and implementation research funded by the US National Institutes of Health, 2005-2012. Nurs Res Pract.

[5] Smits PA, Denis J-L (2014). How research funding agencies support science integration into policy and practice: an international overview. Implement Sci.

[6] Proctor EK, Powell BJ, Baumann AA, Hamilton AM, Santens RL (2012). Writing implementation research grant proposals: ten key ingredients. Implement Sci.

[7] Center for Implementation and Improvement Sciences. Pilot Grant Program; 2016. http://sites.bu.edu/ciis/pilotgrants/. Accessed 2 Nov 2017.

[8] National Institutes of Health. Definitions of criteria and considerations for research project grant (RPG/X01/R01/R03/R21/R33/R34) Critiques | grants.nih.gov. 2016. https://archives.nih.gov/asites/grants/11-14-2016/Grants/peer/critiques/rpg_D.htm. Accessed 26 Oct 2017.

[9] Green J, Thorogood N. Qualitative methods for health research. 3rd ed. Thousand Oaks: SAGE Publications Ltd; 2013.

[10] Hayes AF, Krippendorff K (2007). Answering the call for a standard reliability measure for coding data. Commun Methods Meas.

[11] Krippendorff K (2004). Content analysis: an introduction to its methodology.

[12] R Core (2016). R: a language and environment for statistical computing.

[13] Proctor E, Silmere H, Raghavan R, Hovmand P, Aarons G, Bunger A (2011). Outcomes for implementation research: conceptual distinctions, measurement challenges, and research agenda. Admin Pol Ment Health.

[14] Marsh HW, Jayasinghe UW, Bond NW (2008). Improving the peer-review process for grant applications: reliability, validity, bias, and generalizability. Am Psychol.

[15] Sattler DN, McKnight PE, Naney L, Mathis R (2015). Grant peer review: improving inter-rater reliability with training. PLoS One.

[16] Demicheli V, Di Pietrantonj C. Peer review for improving the quality of grant applications. Cochrane Database Syst Rev. 2007:MR000003. 10.1002/14651858.MR000003.pub2. 10.1002/14651858.MR000003.pub2 PMC8973940 17443627

[17] Jonsson A, Svingby G (2007). The use of scoring rubrics: reliability, validity and educational consequences. Educ Res Rev.

[18] Inouye SK, Fiellin DA (2005). An evidence-based guide to writing grant proposals for clinical research. Ann Intern Med.

[19] Proctor EK, Landsverk J, Aarons G, Chambers D, Glisson C, Mittman B (2009). Implementation research in mental health services: an emerging science with conceptual, methodological, and training challenges. Adm Policy Ment Heal Ment Heal Serv Res.

[20] Proctor EK, Powell BJ, McMillen JC (2013). Implementation strategies: recommendations for specifying and reporting. Implement Sci.

[21] Pinnock H, Barwick M, Carpenter CR, Eldridge S, Grandes G, Griffiths CJ (2017). Standards for reporting implementation studies (StaRI): explanation and elaboration document. BMJ Open.

[22] Oxman AD (2004). Grading quality of evidence and strength of recommendations. BMJ.

[23] Ebell MH, Siwek J, Weiss BD, Woolf SH, Susman J, Ewigman B (2004). Strength of recommendation taxonomy (SORT): a patient-centered approach to grading evidence in the medical literature. Am Fam Physician.

[24] Rycroft-Malone J, Seers K, Titchen A, Harvey G, Kitson A, McCormack B (2004). What counts as evidence in evidence-based practice?. J Adv Nurs.

[25] Rycroft-Malone J, Seers K, Chandler J, Hawkes CA, Crichton N, Allen C (2013). The role of evidence, context, and facilitation in an implementation trial: implications for the development of the PARIHS framework. Implement Sci.

[26] Prasad V, Ioannidis JP. Evidence-based de-implementation for contradicted, unproven, and aspiring healthcare practices. Implement Sci. 2014;9(1) 10.1186/1748-5908-9-1. 10.1186/1748-5908-9-1 PMC3892018 24398253

[27] McCaughey D, Bruning NS (2010). Rationality versus reality: the challenges of evidence-based decision making for health policy makers. Implement Sci.

[28] Bernet AC, Willens DE, Bauer MS (2013). Effectiveness-implementation hybrid designs: implications for quality improvement science. Implement Sci.

[29] Brownson RC, Colditz GA, Dobbins M, Emmons KM, Kerner JF, Padek M (2015). Concocting that magic elixir: successful grant application writing in dissemination and implementation research. Clin Transl Sci.

[30] University of Colorado Implementation Science Program (2018). Ten key ingredients to writing successful d&i research proposals.

